# Effect of different nickel-titanium rotary files on dentinal crack formation during retreatment procedure

**DOI:** 10.15171/joddd.2017.017

**Published:** 2017-06-21

**Authors:** Mehmet Çitak, Taha Özyürek

**Affiliations:** ^1^Department of Endodontics, Faculty of Dentistry, Ordu University, Ordu, Turkey; ^2^Department of Endodontics, Faculty of Dentistry, Ondokuz Mayis University, Samsun, Turkey

**Keywords:** Dentinal crack, ProTaper Next, Retreatment, Reciproc, TF Adaptive

## Abstract

***Background.*** The aim of this study was to compare the dentinal defects caused by Reciproc, TF Adaptive and ProTaper Next NiTi rotary file systems during the retreatment procedure.

***Methods.*** A total of 150 mandibular incisors with straight and single root canals were included in the present study. All the root canals were prepared up to an apical diameter 0.40 mm using stainless steel files. Thirty teeth were randomly stored as the negative control group. A total of 120 specimens were obturated with gutta-percha and AH Plus sealer using vertical compaction technique. Thirty specimens with root canal filling were randomly separated for the only-filled group. Then the teeth were randomly divided into 3 groups; Reciproc, TF Adaptive and ProTaper Next. The retreatment procedure was performed with these NiTi files. Then 150 specimens were cut perpendicular to tooth axis at 3-, 6-, and 9-mm distances from the apex, and examined to determine the presence of any cracks at ×25 under a stereomicroscope. Chi-squared test was used at 5% significance level.

***Results.*** All the tested NiTi file systems were found to cause significantly more dentinal defects compared to unprepared and only-filled groups (P<0.05). No statistically significant differences were found between the groups in terms of dentinal defects (P>0.05). No correlation was found between the slice levels and the dentinal defect distribution (P>0.05).

***Conclusion.*** Within the limitations of present study, all the tested NiTi file systems were found to cause significantly more dentinal defects compared to unprepared and only-filled groups.

## Introduction

Failure following root canal treatment might arise from the continuance of infection due to persistent microorganisms or re-activation of microorganisms in root canal system through coronal or apical path.^1^ An effective retreatment procedure can be available through the removal of existing root canal filling material and then execution of cleaning, shaping and obturation steps.^2^

Currently, the popularity of NiTi rotary file systems has increased because of their superiority over stainless steel files. Thus NiTi files are widely used in root canal preparation and retreatment procedures. Despite many advantages of NiTi rotary file systems, NiTi rotary files were reported to cause dentin defects such as cracks during root canal shaping and retreatment procedures.^3,4^Besides that, it is alleged that the dentin defects, by enlarging, might cause undesired consequences such as vertical root fractures.^5^

ProTaper Next (PTN; Dentsply Maillefer, Ballaigues, Switzerland) is a file system with a horizontal square profile and made of M-Wire alloy. There is a limited number of studies, where this system is used in retreatment procedures.^6,7^ This single file systems made of M-Wire alloy with reciprocation motion has become widely popular recently. Reciproc (RPC; VDW, Munich, Germany), which is one of the most popular single file systems, is also used for retreatment purposes.^6^ Twisted File Adaptive (TFA) (Axis/SybronEndo, Orange, CA, USA) NiTi rotary file system, with its unique motor (Elements Motor; Axis/SybronEndo), aims to combine the advantages of reciprocation and continuous rotation motions. When the file is exposed to any stress (even minimal) within the canal during root canal preparation, it rotates 600° clockwise and then stops and restarts. But, when the file is exposed to stress within the canal, depending on the rate of stress, the Elements Motor modifies the motion up to 370° clockwise and 50° counterclockwise.^8^

In the comprehensive literature review, it was determined that the damage, caused by PRC files on dentin after the retreatment procedure, was examined only in 1 study,^9^ with no studies on TFA and PTN rotary file systems. For this reason, the aim of the present study was to compare the dentinal defects caused by RPC, TFA and PTN NiTi rotary file systems during the retreatment procedure. The null hypothesis of the present study was that there would be no difference between the tested NiTi rotary file systems in terms of the frequency of dentinal defects.

## Methods

### Specimen preparation

A total of 150 mandibular incisor teeth with straight and single root canals and extracted due to periodontal reasons were included in the present study. Then, the soft and hard tissues around the teeth were mechanically removed using a periodontal curette. The crowns of teeth were removed at the cemento-enamel junction under water cooling to achieve a 14-mm root length. The teeth were radiographically examined in mesiodistal and buccolingual directions. The teeth found to have calcification, having a history of previous root canal treatment, involving internal and/or external resorption, or fractured and/or having immature roots were excluded. The selected teeth were then kept in distilled water at 4°C until the experiment.

As in previous studies,^9,10^the tooth roots were coated with aluminum foil and then embedded in acrylic resin (Imicryl, Konya, Turkey).^10^ After the acrylic resin set, the teeth were taken out from the resin, and the foils were removed. For simulating the periodontal ligament, the resin blocks were filled with silicon impression material (Express XT Light Body Quick; 3M ESPE, Neuss, Germany) and the specimens were then placed in the resin blocks again.

#10 K-files (Dentsply Maillefer) were placed in the root canals until the tip was visible at the apex. The working length was set at 1 mm shorter than this length. In all the specimens, the enlargement was performed in accordance with crown-down method, ensuring an apical diameter of 0.40 mm with 2% taper using stainless steel files (Dentsply Maillefer). During the preparation, each specimen was irrigated with 20 mL of 1% NaOCl. A new set of file was used to prepare 4 root canals. Thirty teeth were randomly stored as the negative control group. The remaining 120 specimens were obturated with gutta-percha and AH Plus (Dentsply DeTrey, Konstanz, Germany) sealer using vertical compaction technique. The orifices of the specimens were restored using temporary filling material (Cavit G; 3M ESPE, GmbH, Seefeld, Germany). Then the specimens were kept for 2 weeks at 37°C and 100% humidity for the setting of sealer. Thirty specimens with root canal filling were randomly assigned to the only-filled group. Then the teeth were randomly divided into 3 groups (n=30/each group). All the procedures were executed by a single operator.

### ProTaper Next Group

PTN X3 (30/0.07) and X2 (25/0.06) instruments were used in the crown-down technique. The PTN X3 file was used to remove the gutta-percha and sealer from one-third of the WL, and the X2 file was used in the full WL. The instruments were activated according to manufacturer’s instructions by the torque control endodontic motor (X-Smart, Dentsply Maillefer) at 300 rpm and 2-Ncm torque. The files were used with a brushing motion against the lateral walls. Final apical preparation was then achieved with a PTN X4 (40/0.06) file at the same speed and torque values. The canal was irrigated using a total of 20 mL of 1% NaOCl solution. The final irrigation was performed using 2 mL of 17% EDTA (for 2 minutes) and 1% NaOCl.

### Reciproc Group

The RPC R25 (25/0.08) instrument was used with the VDW Silver Reciproc (VDW, Munich, Germany) endodontic motor using in-and-out pecking motion in the ‘‘RECIPROC ALL’’ program until reaching the WL. The instrument was used with an in-and-out pecking motion. According to the manufacturer’s instructions, gentle apical pressure was applied to the file, and the file was used with a brushing motion. The final apical enlargement was performed with a RPC R40 (40/0.08) file using the same program. The canal was irrigated using a total of 20 mL of 1% NaOCl solution. The final irrigation was performed using 2 mL of 17% EDTA (for 2 minutes) and 1% NaOCl.

### TF Adaptive Group

In this group, TFA ML2 (35/0.06) and ML1 (25/0.08) instruments were used in the crown-down technique as described in the PTN group. The gutta-percha and sealer in the coronal third of the canal were removed using the TFA ML2 file. Then, the TFA ML1 file was used to reach the WL. The instruments were activated with the Elements Motor in the ‘‘TF Adaptive’’ program. The final apical enlargement was performed with a TFA ML2 (35/0.06) file using the same program. The canal was irrigated using a total of 20 mL of 1% NaOCl solution. The final irrigation was performed using 2 mL of 17% EDTA (for 2 minutes) and 1% NaOCl.

### Sectioning and microscopic examination

**Figure 1:**
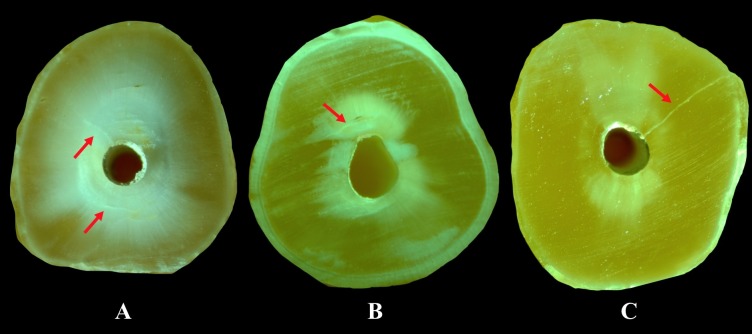
The samples showing dentinal defects (red arrows). A; Twisted File Adaptive B; ProTaper Next C; Reciproc.

Under water cooling using a low-speed saw (Isomet; Buehler Ltd, Lake Bluff, IL, USA), the roots of 150 specimens were cut perpendicular to the tooth axis at 3-, 6-, and 9-mm distances from the apex, and 3 slices were obtained from each specimen. The digital images of the slices were taken under ×25 magnification using a digital camera connected to a stereomicroscope (Olympus BX43, Olympus Co., Tokyo, Japan). A total of 450 digital images (90 images in each group) were examined for the presence of any cracks. To define crack formation, 2 different categories were made (i.e., “no crack” and “crack”) to avoid the confusing description of root cracks. “No crack” was defined as root dentin without cracks or craze lines either at the internal surface of the root canal wall or the external surface of the root. “Crack” was defined as all the lines observed on the slice that either extended from the root canal lumen to the dentin or from the outer root surface into the dentin (Figure 1).^11^

### Statistical analysis

In intergroup statistical analysis of the dentinal defect incidence, chi-squared test was employed. The level of statistical significance was set at 5%. Moreover, in order to determine the distribution of dentinal defects by the slices (3, 6, 9 mm), Pearson’s correlation test was used. All the statistical analyses were performed using SPSS 21 (IBM-SPSS Inc., Chicago, IL, USA).

## Results

**Figure 2:**
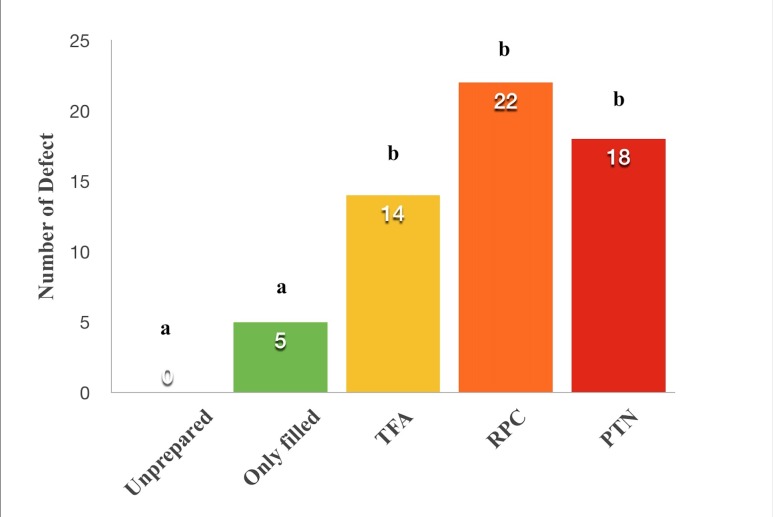
The number of teeth exhibiting dentinal defects after retreatment procedures. Groups with different letters are statistically significant at P<0.05.

**Figure 3:**
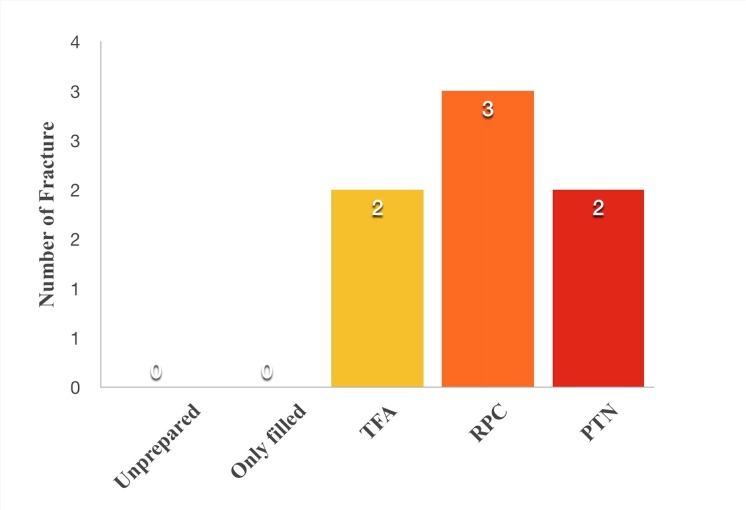
The number of vertical root fractures after retreatment procedures. There were no statistically significant differences between the groups at P>0 .05.

The distribution of dentinal defects by the groups is shown in Figure 2. The distribution of vertical fractures in the groups is presented in Figure 3. All the tested NiTi file systems were found to cause significantly more dentinal defect than unprepared and only-filled groups (P<0.05). No statistically significant differences were found between the groups in terms of the dentinal defects (P>0.05). No correlation was found between the slice levels and the dentinal defect distribution (P>0.05). No statistically significant differences were observed between the groups in terms of the vertical fractures caused by NiTi rotary file systems (P>0.05).

## Discussion

The vertical root fracture in endodontically treated teeth is an unfavorable problem, and usually results in the loss of teeth.^12^For this reason, it was aimed in the present study to examine the dentinal defects caused by PTN, RPC and TFA NiTi rotary file systems, which have different kinematics, on the dentin during retreatment procedures. According to the results of the present study, no significant difference was found between the NiTi rotary file systems in terms of the incidence of dentinal defects. Thus, the null hypothesis was accepted.

Because of the smaller apical diameters, the mandibular incisor teeth were used in the present study. Before being included in the study, the teeth were examined under a stereomicroscope for the presence of any fractures or cracks. However, there might have been cracks in some of the teeth, not visible externally. The absence of any dentinal defect in the negative control group in the present study indicates that the teeth were free of cracks. The absence of any dentinal defects in the negative control group is consistent with other studies.^13,14^ In previous studies, it was reported that dentinal defects occurred during root canal preparation with NiTi rotary file systems.^13,15^ In contrast, it was also reported that no dentinal defects occurred during root canal preparation with hand files.^3,16^ For this reason, the crown-down technique was used for root canal preparation with the use of hand files, ensuring an apical diameter of 0.40 mm.

In the present study, the teeth were cut perpendicularly to the long axis, and then analyzed under a stereomicroscope. Even though the slicing method is a method damaging the specimens, no dentinal defect was observed on the specimens in the negative control group. Shemesh et al^17^ examined the efficiency of optical coherence tomography in determining vertical root fractures. Researchers reported that the optical coherence tomography was very useful in detecting vertical root fractures, and that it successfully showed at which point vertical root fractures were localized. De-Deus et al^18^ examined the dentinal defects caused by various NiTi file systems by using computerized micro-tomography. Researchers reported that computerized micro-tomography is a very useful and conservative method for determining dentinal defects. However, the computerized micro-tomography’s capability of determining small dentinal defects is not clear. If the resolution of computerized micro-tomography is larger than the dentinal defect, then it could not detect the defect. For this reason, the slicing method was used in the present study, as in previous studies.^3,13,19^

NiTi file manufacturers generally recommend using the file on a single tooth. In the present study, in order to prevent the effects of deformation of files on the results, the preparation of 4-canal first maxillary molar teeth was taken as base, and the files were replaced after using them in 4 canals.^14,20^In previous studies, the increased apical diameter in retreatment procedure was reported to decrease the amount of residual root canal filling material.^21,22^However, since the NiTi files’ efficiency in forming dentinal cracks was tested in the present study rather than the efficiency in removing root canal filling material, the apical diameter was not increased. In previous studies, Gates-Glidden drills were reported to remove large amounts of dentin from the root canals and to cause dentinal defects.^23,24^For this reason, in order to avoid such effects in the present study, Gates-Glidden drills were not used to remove gutta-percha from the coronal third.

Studies have reported that high concentration of NaOCl solution decreases the elastic modulus and hardness of dentin.^25,26^In the present study, 1% NaOCl solution was used as an irrigation solution for protecting the microstructure of dentin. Thus, it was aimed to ensure that the dentinal defects were mainly related with the mechanical preparation. In the present study, the teeth were obturated in accordance with the vertical compaction method. For this reason, contrary to previous studies^8,19^in which lateral compaction technique was used for obturation, the dentinal defects were observed in the only-filled group.

Since there is no study available on the dentinal defects caused by TFA and PTN NiTi rotary file systems after the removal of root canal filling material, the results of presents study cannot be directly compared to other studies. According to the results of the present study, it was determined that the amount of dentinal defect caused by NiTi rotary files was higher than the amount in unprepared and only-filled groups. This result can be explained with the root canal filling material removal procedures during retreatment. Moreover, the results of the present study are consistent with those of Shemesh et al,^27^indicating a lower amount of dentinal defects in the only-filled group when compared to the retreatment group. In their study on dentinal defects after the retreatment procedure, Üstün et al^8^corroborated the results of the present study and reported no significant differences between ProTaper Universal Retreatment (Dentsply Maillefer) and RPC rotary NiTi file systems.

It has been reported that the characteristics of NiTi files such as the tip design, cross-section, or variable or constant taper might affect the formation of dentinal defects.^13,16^Topcuoglu et al^19^examined the dentinal defects occurring after retreatment using NiTi rotary file systems and hand files, reporting that all the systems created dentinal defects, with no statistically significant differences between the groups. The authors stated that the design properties of NiTi files have no effect on the formation of dentinal defects. The results of the present study showed no relationship between the design and movement kinematics of the tested files and the dentinal defects occurring during the removal of root canal filling material.

Despite attempts to simulate the clinical conditions in the laboratory environment, external factors such as storage of teeth until the slicing phase after the extraction might have affected the results of the study, especially in studies examining the mechanical properties of teeth.^15^

## Conclusion

Within the limitations of present study, it was concluded that all the tested NiTi file systems caused significantly more dentinal defect compared to the unprepared and only-filled groups.

## Acknowledgments

The authors deny any conflicts of interest related to this study.

## Authors’ contributions

MÇ & TÖ equally contributed to the design and the execution of the study, as well as the interpretation of the findings. Both authors have read and approved the final manuscript.

## Funding

The study protocol was funded by Ondokuz Mayis University.

## Competing interests

The authors declare no competing interests with regards to the authorship and/or publication of this article.

## Ethics approval

The study protocol was approved by the ethics committee of Ondokuz Mayis University.
